# Nobel somatosensations and pain

**DOI:** 10.1007/s00424-022-02667-x

**Published:** 2022-02-14

**Authors:** Peter W. Reeh, Michael J. M. Fischer

**Affiliations:** 1 Institute of Physiology and Pathophysiology, University of Erlangen-Nürnberg, Universitätsstrasse 17, 91052 Erlangen, Germany; 2 Center for Physiology and Pharmacology, Medical University of Vienna, Schwarzspanierstrasse 17, 1090 Vienna, Austria

**Keywords:** 50 ion channels, GPCRs, Agonists, Antagonists

## Abstract

The Nobel prices 2021 for Physiology and Medicine have been awarded to David Julius and Ardem Patapoutian "for their discoveries of receptors for temperature and touch", TRPV1 and PIEZO1/2. The present review tells the past history of the capsaicin receptor, covers further selected TRP channels, TRPA1 in particular, and deals with mechanosensitivity in general and mechanical hyperalgesia in particular. Other achievements of the laureates and translational aspects of their work are shortly treated.

Two days after the publication in Nature of the paper from David Julius’ lab, that finally led to this year’s Nobel Price for Physiology and Medicine, one could see many (white) people with black noses strolling through the rows of posters at the huge US-American Neuroscience Meeting in New Orleans 1997. The publisher had provided a large number of offprints with the black Nature title page depicting a variety of hot chili peppers, but they were quickly out of stock so that a second load of all too freshly printed offprints was supplied. Excited scientists grabbed the paper and curiously rubbed their noses while absorbing the spectacular news on the ‘Red-hot receptor’ [33].

## Past history

The enormous interest in capsaicin and the successful cloning of its receptor channel had exponentially grown since middle of the 1970s. At that time, the groundbreaking works starting in the 1940s on capsaicin of Miclos (Nicholas) Jancso, his wife Aurelia Jancso-Gabor, later his son Gabor Jancso, and his early collaborator, the late Janos Szolcsanyi, had become widely appreciated through publications in English [81, 164]. It was the incomparable—by that time—selectivity of capsaicin’s actions that inspired the pain research community: It did not smell, it did not taste, not numb nor damage the oral mucosa but just hurt, inducing the well-known burning pain sensation of Habanero chili. In scientific terms, it did exclusively excite a large and distinct subpopulation of nociceptors anywhere in the mammalian body, but not in avians or amphibians [83]. If concentration and duration of capsaicin application were large enough, the excitation was followed by a sustained insensitivity to heat, but not mechanical, and to chemical (algogenic) stimulation, e.g. by inflammatory mediators like bradykinin or histamine. When capsaicin was systemically administered in rodents or directly applied to nerve endings, peripheral nerve fibres or sensory neurons a lasting, highly selective neurotoxic effect with degeneration and necrosis was achieved that early awakened hopes for a therapeutic utilization [19]. Decades later, Qutenza^TM^ was approved, a plaster delivering capsaicin to painful neuropathic skin, and is still the only approved direct TRP channel modulator [113]. Promising appears the soon to be expected approval of CNTX-4975, an ultrapure formulation of trans-capsaicin to be injected into osteoarthritic joints [157]. A long tradition has the desensitization of the hyperactive (‘neurogenic’) urinary bladder by capsaicin instillation [42].

## Capsaicin receptor TRPV1

**Fig. 1:**
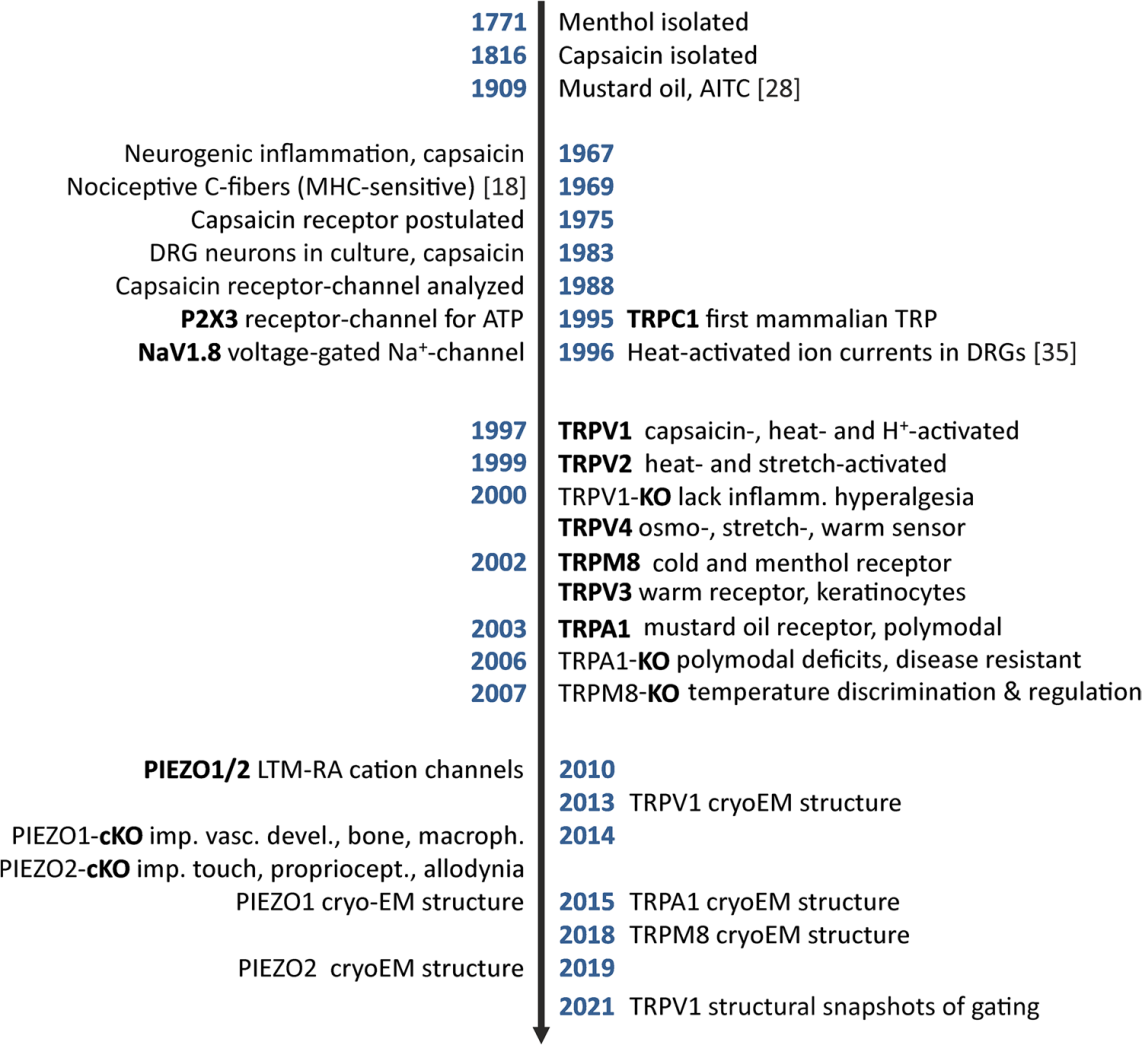
Timeline of milestones. References mentioned only here: [18, 28, 35]

Based on the then accumulated pharmacological evidence, already 1975 the existence of a capsaicin receptor was postulated [165]. With the advent of rat sensory neuron cultivation, intracellular recording indicated that capsaicin—chemically a vanilloid—activates an ionotropic receptor, a depolarizing, excitatory ion channel which was soon analyzed in detail by patch-clamp techniques and shown to be an unselective cation conductor [10, 182]. It is not just the ‘cherry on the cake’ that the capsaicin receptor *transient receptor potential vanilloid 1* (TRPV1) was finally cloned from rodent sensory neurons, employing an elegant search technique, but this achievement released an avalanche of other TRP channel discoveries with today (Nov. 2021) more than 19,000 papers in PubMed [188]. These penetrate almost all fields of physiology and medicine, including so diverse disciplines as diabetology, cardiology, or oncology. David Julius and his lab at UC San Francisco contributed at least 48 major publications to this flood of papers. Milestones in the timeline are depicted in Fig. 1.

The cloned and heterologously transfected TRPV1 met all functional expectations: high calcium ion permeability, Ca^2+^-dependent desensitization and cytotoxicity, specific expression in spinal and trigeminal sensory ganglia, and activation by noxious heat [33]. Only one property required an early second paper to demonstrate the activation of TRPV1 by tissue acidosis as in inflammation, tumours, and ischemic muscle work [169]. This important source of pain can be blocked by an experimental TRPV1 antagonist (BCTC) in a human psychophysical model [69]. Loss of sensory neuronal proton sensing and of inflammatory hyperalgesia was to be predicted from the phenotype of TRPV1 ‘knockout’ mice [31, 44]. However, the reduction of behavioural heat sensitivity was marginal in these knockout animals. Only recently it was shown that in mice three different genes for heat-activated ion channels, TRPV1, TRPA1, and TRPM3, need to be deleted in order to abolish noxious heat avoidance [173]. In clinical trials, it was therefore surprising that some of the developed TRPV1 inhibitors abrogated heat sensing in patients to an extent that burn or scalding injuries were to be feared. However, translational research on sensory neurons and humans (psychophysics) recently confirmed this loss of heat sensing after topical capsaicin-induced desensitization of the skin [144]. Although the TRPV1 antagonists were efficacious analgesics, e.g. in osteoarthritic patients, these drugs were not pursued, also because they transiently increased body temperature [91]*.* Other approaches, leaving the native TRPV1 intact but trying to counteract its sensitisation during inflammation, have unfortunately not been further developed [54, 65, 155]. One new point of attack has recently been proposed: The high calcium conductance of TRPV1 leads to activation of the Ca^2+^-activated Cl^−^ channel ANO1 co-expressed in nociceptive neurons which amplifies the depolarizing action and action potential discharge; this interaction could be pharmacologically targeted [167].

## Integrator and sensitizer TRPV1

However, scrutinizing the TRPV1 homotetramer with single-particle cryo-electron microscopy revealed potential allosteric activation mechanisms and ligand binding sites that may in the future allow to develop targeted small molecules to interfere with one but not the other function of the polymodal receptor channel [27, 97]. The cryo-electron microscopy and related studies also helped to better understand the paramount capacity of TRPV1 to integrate a multiplicity of endogenous (‘endovanilloids’) and exogenous stimuli by enabling synergistic or sensitizing actions of the different signals. Much of this capacity is based on the action of phospholipase C (PLC)—a common activator of many TRP channels—to remove the double-edged effectors phosphatidylinositol-4,5-bisphosphate (PIP_2_) and phosphatidylinositol 4-phosphate (PI4P) from the membrane domain hosting TRPV1 [26, 64]. Many G protein–coupled receptors such as the B2 receptor for bradykinin activate PLC and also disinhibit TRPV1. In addition, diacylglycerol (DAG), resulting from PIP2 cleavage by PLC, activates protein kinase C which phosphorylates and further promotes TRPV1 activation. This cascade of events can, for example, lower the heat threshold of rat skin nociceptors as far as below body temperature, thus likely producing inflammatory pain [96]. Also protein kinase A, activated through prostaglandin receptors, can sensitize TRPV1 which may be the reason why the COX inhibitor ibuprofen, orally or topically administered, strongly reduced the pain from experimental tissue acidosis in the skin and muscle in the above-mentioned human model [156]. Later, cryo-EM investigations using lipid nanodiscs generated snapshots of TRPV1 in interim states, providing a glimpse at how gating works [186].

## An earlier TRP and other ‘pain-related’ channels

TRPV1 was not the very first ‘pain-related’ ion channel to be cloned, though particularly seminal. John Wood and his lab had previously discovered the P2X3 receptor channel for extracellular ATP and diadenosine polyphosphates (‘alarmones’) which are cellular stress signals [36]. In 1996, they had cloned the TTX-resistant voltage-gated sodium channel NaV1.8 which turned out indispensable in nociceptors for action potential generation and encoding of discharge rate at noxious cold and hot temperatures [3, 170, 189]. TRPV1 was also not the very first TRP channel to be cloned, as Craig Montell’s lab (Johns Hopkins, Baltimore, at the time) had previously discovered TRPC1, the human homolog of the *drosophila* mutant Ca^2+^ channel involved in phototransduction that had led to the misnomer *transient receptor channel* (TRP) [178]. In fact, TRPV1 behaves only ‘transient’, i.e. desensitizing, inactivating during ongoing stimulation, if Ca^2+^ entry through the channel is not prevented by removing and adequately buffering extracellular calcium [33]. A recent review article provides an overview of the TRP channel superfamily and its 28 mammalian members [171].

## TRPV2

The big wave of TRP channel discoveries gained momentum 1999 with the cloning by David Julius’ lab of the rodent TRPV2, capsaicin insensitive but still named ‘V’ for its similarity to TRPV1. TRPV2 first appeared as a heat transducer for temperatures > 50° C, but global knockouts showed no thermo- or mechanosensory deficits even in inflamed skin [32, 126]. However, sensory neuron-specific conditional TRPV2 knockout mice were hyposensitive to noxious pressure and a distinct subpopulation of their sensory neurons lacked stretch-activated Ca^2+^ influx [85]. Stretch activation and high expression of TRPV2 in cardiomyoctes prompted clinical studies in dilated cardiomyopathy [80]. TRPV2 also plays a role in innate immunity by its expression in macrophages which require the channel for efficient phagocytosis. Like many TRP channels, TRPV2 is redox sensitive, even the human homolog, originally insensitive to heat, gains heat sensitivity with thresholds below body temperature when oxidized, and, vice versa, macrophages reduce their phagocytic activity when exposed to reducing agents [57].

## TRPV4

TRPV4 was cloned in 2000 as an osmoreceptor for hypotonicity and putative stretch receptor but soon shown also to serve as a transducer for warm ambient temperatures [62, 98]. TRPV4 was the first TRP channel mutations of which were associated with various genetic diseases in humans ranging from asthma over skeletal dysplasias to neuropathy; global knockout mice show deficits in various visceral pain models [179]. The complex phenomenologies are due to a very widespread expression of TRPV4 in many cell types and organs, i.e. in epithelial cells, particularly keratinocytes [91]. A stunning cascade of events leading to cholestatic itch in liver diseases has recently been established: Enhanced blood and skin levels of lysophosphatidylcholine (LPC) activate TRPV4 in keratinocytes which in response release the particular microRNA-146a known to act as an inflammatory mediator in the innate immune system. This acts probably through *toll-like receptor 7* (TLR7) which finally activates TRPV1 in nociceptors, some of which also act as pruriceptors inducing itch [37].

## Price worthy TRPs, TRPV3 and TRPM8

In 2002, the second Nobel laureate Ardem Patapoutian entered the stage of TRP channel discoveries. TRPV3 was cloned in his lab at the Genomics Institute of the Novartis Research Foundation in La Jolla/San Diego, adjacent to the Scripps Research Institute where he moved soon after [131]. TRPV3, activated by warm temperatures (> 32° C), was abundantly expressed in skin keratinocytes, not in rodent but in human sensory (DRG) neurons, and accordingly, from knockout mice no alterations in thermal preference and noxious heat withdrawal were reported [79]. TRPV3 found great attention in dermatology, a human gain-of-function mutation is in part responsible for the hyperkeratotic and mutilating Olmsted syndrome; TRPV3 plays roles in the control of hair growth and lipid secretion, in atopic dermatitis and pruritus [91, 107]. However, by optogenetic inhibition of keratinocytes in mice, it was recently shown that stimulated ATP release from these cells contributes to behavioural heat, cold, and mechanosensitivity through neuronal P2X4 purinoceptor channels [109, 147], and TRPV3 expression is essential for the heat-induced ATP release that is able to activate nearby DRG neurons in co-culture with keratinocytes [103].

Also 2002, the cold transducer and menthol receptor TRPM8 was cloned in Patapoutian’s and, independently, in Julius’ lab [106, 131]. TRPM8 is particular among the TRPs, as it is alone largely responsible for the delicate sense of cooling, being expressed in the ‘cold’ fibres, unmyelinated C-fibres in rodents and thinly myelinated in humans. Due to the widely overlapping stimulus-response curves of ‘warm’ and cold fibres around body temperature, TRPM8 indirectly also contributes to the equally delicate sense of warming. Ongoing activity in cold fibres and their input to the CNS appear to inhibit the throughput of warm fibre activity; temperature increase immediately silences the cold fibres and, thus, enables the perception of warming [125]. Accordingly, TRPM8 knockouts cannot discriminate between warm and cool stimuli; in addition, they do not exhibit inflammatory or neuropathic cold allodynia, a painful sequelae of cancer chemotherapies [12]. These knockout mice also tend to develop obesity due to day-time hyperphagia; and ageing wildtypes, showing reduced TRPM8 expression and function, cannot properly prevent heat loss by vasoconstriction in cool environment [141, 168]. Vice versa, TRPM8 agonist treatment in wildtypes leads to enhanced energy expenditure and loss of body weight [39]. The inhibitory action of TRPM8 expressing cold fibres seems to extend to nociception: Hyperalgesia in neuropathy and inflammatory models was antagonized by the TRPM8 agonists menthol or icilin at the affected skin or upon intrathecal administration; these analgesic effects were lost after specific knockdown of TRPM8 by intrathecal antisense oligonucleotides [134]. Moreover, a human population identified by single nucleotide polymorphism in or near the TRPM8 gene has been found to carry a reduced risk of migraine headaches. Carriers also show a reduced expression of TRPM8 in DRGs (post mortem) and, in vivo, a correlating reduction of cold and cold pain sensitivity [59]. These findings justify the clinical studies with menthol and various TRPM8 antagonists against pain and itch [91]. A particular role of TRPM8-expressing nerve fibres in the cornea has been identified: When the tear film on the eye evaporates, the temperature drops and the osmolality increases; both effects are adequate stimuli to TRPM8 and lead to reflex blinking and lacrimation [127, 136]. The structure of TRPM8 has been discovered by cryo-EM [184].

## TRPA1, almost universal chemosensor and polymodal receptor

Although an ankyrin-rich TRP-like protein was first identified in 1999 in human fibroblasts, its huge potential as ANKTM1, soon renamed TRPA1, came to appearance in 2003 with its cloning in Patapoutian’s lab. First described as a noxious cold transducer, its role as a chemosensor for many pungent plant compounds such as the volatile mustard oil ingredient allyl-isothiocyanate (AITC, e.g. in horseradish) was soon recognized and its function as an effector ion channel for bradykinin and products of phospholipase activation such as arachidonic acid and diacylglycerol [11, 158]. David Julius’ lab soon joined in, extending the library of TRPA1 agonists to other isothiocyanates and, surprisingly, to delta(9)-tetrahydrocannabinol (THC) that was already in fashion as a would-be analgesic and now turned out to be a potentially painful irritant. In addition, they confirmed the TRPA1 activation by G protein–mediated phospholipase C (PLC) activation and provided first evidence for TRPA1 gating by intracellular calcium ions [82]. Meanwhile, the binding pocket in TRPA1 for intracellular Ca^2+^ ions, accounting for activation, desensitization, and metabotropic modulation, has been identified by cryo-EM analysis and the mechanisms of cysteine-dependent activation by intracellular electrophilic compounds as AITC have further been elucidated [187]. The latter involves a most unusual covalent, but not irreversible, ligand binding principle, which can lead to the formation of activating disulphide bridges between adjacent cysteine thiols. This applies, for example, to methylglyoxal, a cytotoxic side product of glycolysis, accumulating in diabetic or uremic patients and contributing to their potential development of painful neuropathy [48]. Methylglyoxal belongs to the highly reactive endogenous carbonyl species (RCS) that are TRPA1 agonists as well as the reactive oxygen and nitrogen species (ROS, RNS) such as H_2_O_2_ and peroxynitrite [6, 8]. However, H_2_O_2_ is used in molar concentration to clean and disinfect wounds which does not hurt, in contrast to the TRPV1 agonist ethanol. Reason for the discrepancy is that many of the reactive species, in particular H_2_O_2_, are relatively impermeable and therefore much more potent at oxidizing proteins like TRPA1 if generated, or experimentally applied, intracellularly. An example is again methylglyoxal which is overproduced in the insulin-independent neurons under the substrate pressure of hyperglycaemic episodes in diabetes, potentially evoking pain [7]. Other examples are Fe^2+^ ions as in heme which potentiate H_2_O_2_ activation of TRPA1 by catalytic formation of hydroxyl radical (OH^.^), or the heme precursor protoporphyrin IX, a chromophore present in all cell types, which generates singlet oxygen (^1^O_2_) activating TRPA1 under the influence of violet visible light (406 nm). This causes pain when unpigmented human skin is irradiated, which serves as a model for the painful photosensitivity of porphyria patients [9, 72]. Near ultraviolet light (UVA) could also act through creating oxidative stress, but in human melanocytes it is a UVA-induced, retinal- and opsin-dependent activation of PLC that activates TRPA1, channelling Ca^2+^ ions which stimulate the cellular melanin synthesis [16]. This is a striking example for the widespread extraneuronal TRPA1 expression, particularly in epithelial cells, which makes the development of TRPA1 blockers for clinical use so difficult and lengthy, although preclinical results are most promising. On the other hand, epigenetic regulation of expression of TRP channels in diseases and overexpression in certain tumours may open new therapeutic options [70, 91].

TRPA1 is the most polymodal receptor channel of all TRPs, activated or inhibited by hundreds of natural and synthetic chemicals including common drugs such as dipyrone (metamizol) and acetaminophen (paracetamol), the latter accounting for analgesic and antipyretic effects [61, 77, 119, 149], and somehow contributes to noxious heat and cold transduction [74, 110, 154]. Particular chemical cases are cigarette smoke and its nicotine-free gaseous phase, tissue acidosis activating only the human TRPA1 but not rodent and non-human primate homologs, and nitroxyl anion (H^+^NO^−^) which results from the chemically unusual interaction of the two gasotransmitters H_2_S and NO. [47, 87, 142].

## TRPA1 channelopathy and other diseases

A peculiar human TRP channelopathy discovered in members of a Colombian family is a gain-of-function mutation in the TRPA1 gene that leads to severe upper body musculoskeletal pain attacks when fatigue, exhaustion, hunger coincide with a chilly environment [92]. This **f**amilial **e**pisodic **p**ain **s**yndrome (FEPS1) and its very particular characteristics may result from an inhomogeneous composition of the TRPA1 tetramer, consisting of wildtype and mutant monomers in various combinations [117]. Among the preclinical disease models, TRPA1 is involved in almost all painful or pruritic disorders, prominently in respiratory (cough), pancreatic, and inflammatory bowel disease (IBD) models [70, 91]. In three models of chemically induced colitis (TNBS, DSS, oxazolone), TRPA1 gene deletion or pharmacological block prevented or cured the disease. In addition, the TRPA1-mediated (via Ca^2+^ influx) neuropeptide release, of substance P (SP) in particular, was identified as the decisive disease promoter; disruption of SP signalling also had preventive or curative effects [50–52]. These findings, including the detrimental role of SP, were soon corroborated in pancreatitis models [34]. Nonetheless, the gastrointestinal research field is still controversial with respect to TRP involvement, also because none of the disease models is fully accepted to reproduce the human diseases [43]*.* Part of the controversy resulted from the use of capsazepine, a classical capsaicin antagonist developed long before TRPV1 was cloned; the much-used drug tool was reported to exert curative effects in models of colitis and pancreatitis [58, 180]. However, it then turned out that these beneficial effects were the same in global TRPV1 knockouts, and that capsazepine actually acted as a weak TRPA1 agonist with strong desensitizing aftereffects [90]. Systemically administered (in drinking water) capsazepine even achieved a body-wide desensitization against noxious heat stimuli and chemical irritants in wildtype mice but not TRPA1 knockouts—much like systemic capsaicin (or resiniferatoxin) would do, reflecting the co-expression of TRPA1 and TRPV1 in sensory neurons and their cross-desensitizing effects [89]*.*

## TRPA1 and other mechanosensitivities

The direct and indirect mechanosensitivity of TRPA1 completes the picture of TRPA1 polymodality. Its mechanosensitivity was discovered in David Corey’s lab where global knockouts had been generated that showed deficits in sensing ice cold, chemical, and punctate mechanical stimuli [94]. Ardem Patapoutian’s and a collaborating lab soon joined in demonstrating mechanical activation of TRPA1 first in *C. elegans* and then in mice where it contributes to inflammatory hyperalgesia [88, 132]. Recently, the inherent responsiveness to graded (negative) pressure of the purified hTRPA1 protein reconstituted in artificial lipid bilayer has been established by single-channel patch-clamp recordings [111]. A hint for the mechanism of activation comes from lipopolysaccharides, toxic decay products of Gram-negative bacteria that activate TRPA1 without being able to bind to the receptor. Instead they integrate into the outer leaflet of the neuronal membrane, distorting it and exerting ‘force-from-lipid’ on the transducer channel [108]. This concept had been introduced in 2004 and explained by ‘hydrophobic mismatch’ between lipophilic amino acid helices of the transmembrane components of stretch-activated ion channels and the surrounding lipid bilayer [159]. The authors employed a tarantula toxin (GsMTx4) that inhibited mechanosensitive TRPC1, TRPC6, and other stretch-activated ion channels but later was shown to also activate TRPA1 potently [71]. A stunning example for the versatile application of mechanosensitivity in evolution is *Drosophila* vision where stretch activation of TRP and TRPL ion channels by force-from-lipid is the final step in the ‘photomechanical’ transduction cascade that leads to measurable contraction of the omatidium [68].

Mechanical hyperalgesia is the main reason for chronic pain in daily life, e.g. in osteoarthritis. One reason why the mechanisms of painfully exaggerated mechanosensitivity are not fully unravelled is that so many functional proteins show mechanosensitive properties, one way or the other. Not only ion channels and G protein–coupled receptors but even the ubiquitous phospholipases are mechanosensitive due to their ‘interfacial activation’, enhancing the catalytic activity 1000-fold upon contact of the enzymes with the inner leaflet of the plasmalemma which is induced by increased membrane tension, heating, submicromolar Ca^2+^ concentrations, or ERK/MAPkinase signalling [115]. This mechanism may be involved in the dramatic increase of prostaglandin E2 release from the mouse colon upon distension by intraluminal pressure. The same stimulation also leads to a graded release of vasoactive neuropeptides (CGRP and SP, ‘neurogenic inflammation’) from colonic primary afferents and, in vivo and in parallel, to a measurable (iEMG) muscular defence reaction in the abdominal wall [114, 145]. Both ‘visceromotor’ response and CGRP release upon colonic distension depend largely and to about equal degrees on TRPV4 and TRPA1 expression and can pharmacologically be diminished by selective inhibitors. However, not every neuronal membrane equipped with TRPA1 is mechanosensitive. Nociceptive nerve fibres in peripheral nerves do express TRPA1 as well as TRPV1 in their axolemma and respond to AITC as well as to capsaicin and heat, just like their nerve endings in the skin, but they are not sensitive to pressure, not even to forces much higher than required to excite their polymodal terminals [17, 75, 177]. This selective suppression of one but not the other modality could possibly be due to equally mechanosensitive antagonists such as the two-pore domain K^+^ channels (K2P) TRAAK and TREK1 that could counteract the depolarization by TRPA1 by hyperpolarizing currents. Their activation by force-from-lipid is well established [23, 24]. Both K2P channels also play a role in the fine adjustment of noxious heat and cold sensitivity, counteracting TRPV1 and TRPM8 [120].

Unravelling mechanosensitivity has recently been further complicated by the optogenetic demonstration that Schwann cell processes, joining sensory nerve terminals into the epidermis, transduce and transmit not only heat and cold but also mechanical stimuli, evoking pain-related behaviours in mice [1]. The transducers and transmitter of the Schwann cells have not yet been identified, but the findings remind of the above-mentioned TRPV3 and TRPA1 expression in keratinocytes that confers the same sensory capacities on cells that are also in close contact with nociceptive nerve endings in the skin [147]; as a possible transmitter, ATP had previously been proposed [103]. In addition, PIEZO1 and PIEZO2 have recently been found in keratinocytes and in Schwann cells, respectively [76, 153].

## Nobel-prized PIEZOs

In view of the confusing multiplicity of cell types showing mechanosensitivity and the large variety of possible mechanisms, the discovery in Ardem Patapoutian’s lab of the PIEZO channels feels like the revelation of a great unifying concept [41]. Indeed, they account for a whole range of low-threshold mechanosensitivities in various cell types and sensory nerve terminals. Like expression cloning for TRPV1, also identification of the PIEZOs required persistence for a brute force approach on mouse Neuro2A neuroblastoma cells which showed constitutive mechanosensitivity [123]. Candidate genes with transmembrane domains were addressed one-by-one by means of RNA silencing until the mechanoreceptor, encoded by the Fam38A gene, was found. The last one of 72 candidates brought this success. The receptor was named PIEZO1, derived from πιέζω the Greek word for exerting pressure. In hindsight, there are also Greek words for touch or stretch, but the existence of technical piezoelectric sensors, which convert movement into voltage, was suggestive. PIEZO1 is sufficient to endow HEK293t cells with a mechanically induced unselective cation current in response to perturbation of the lipid bilayer [45]. Its molecular size as well as 38 transmembrane domains by far exceeded known ion channel families at the time. Although PIEZO1 expression is reported from sensory neurons, [143], the important role is played by its only known sibling, PIEZO2, which was identified by sequence homology. The latter is not only present in DRG neurons but also expressed in the cutaneous Merkel cells that form a functional complex (‘touch dome’) with the terminals of a fast conducting Aβ-fibre; together they constitute an exquisitely sensitive tactile receptor [181]. Mice with a conditional deletion in DRGs of the gene for PIEZO2 showed severe deficits in proprioception and cutaneous mechanosensitivity [139], much like patients with a lack-of-function mutation who exhibit ataxia and dysmetria in addition to a lack of interoception and discriminative touch perception [38].

## PIEZOs beyond somatosensation

With the establishment of PIEZO2 in mechanosensation, Ardem Patapoutian focussed on other body sites. This sparked a remarkable set of so far 30 publications alone from his laboratory, and occurrences in far more functions than expected. PIEZO2 is found in the vagal stretch receptors of the tracheobronchial wall and is important for regular breathing in neonatal life and later for the Hering-Breuer lung inflation reflex that ends inspiration and initiates expiration and bronchial relaxation [121]. Enterochromaffin cells in the gastrointestinal tract are mechanosensitive through PIEZO2 and this regulates serotonin release controlling secretion and motility [5]. In the bladder, PIEZO2 is found in urothelial cells as well as in the innervating afferent neurons subserving urinary function [105]. As previously mentioned for the skin, the dual appearance in neuronal and non-neuronal cells required targeted deletions to conclude on the relative importance of the receptor in a particular cell type.

PIEZO1 can keep up with the multiplicity of diverse functions of PIEZO2. PIEZO1 in endothelial cells is essential for vascular development and remodelling due to its sensitivity for blood flow–induced shear stress [138]. Red blood cells require PIEZO1 to cope with the mechanical stress when passing narrow capillaries [25]; rare mutations in the gene lead to different syndromes causing haemolytic anaemia in patients [4]. A PIEZO1 gain-of-function variant with about 30% prevalence in Africa was shown to reduce red blood cells size and to increase resistance against Plasmodium falciparum infection [101]; whether the same variant is of importance for glaucoma is less clear [13]. This variant also shows a role of PIEZO1 in upregulating phagocytic activity of macrophages which leads to increased erythrocyte turnover and elevated plasma iron levels [102]. PIEZO1 in osteoblasts plays a role in bone homeostasis and remodelling; the conditional deletion of the channel leads to reduced bone mass and fractures [174]. This seems to be mediated by an effect of PIEZO1 on developmental fate in the osteoblastic lineage [160].

A major step forward was the discovery of a chemical agonist for PIEZO1, the first called Yoda1 [162], followed by the chemically different Jedi1/Jedi2 [176], demonstrating druggability, but still with limited potency. These substances do not activate PIEZO2, for which no agonist has been discovered. The engineering of chimeras between PIEZO1 and PIEZO2 helped to identify the PIEZO1 binding sites for these agonists [95]. An important leap was solving the cryo-EM structures of PIEZO1 [60, 63, 148], and PIEZO2 [175]. The receptor channels are trimers (> 1 MegaDalton) with large propeller-like blades around an extracellular ‘nano-dome’, which generate a large in-plane membrane area expansion. Deformation of the latter opens the pore, but more work is still required to elucidate the molecular mechanics [166].

## PIEZO2 and pain

The Nobel committee’s press release hardly mentioned the word pain in conjunction with PIEZO2, although mechanically induced pain and hyperalgesia under the physical loads of daily life are a scourge of humanity with a large unmet medical need. However, PIEZO2 does seem to be involved in pain, although mainly in the particular pathophysiology of tactile allodynia, painful sensations evoked by touching or brushing affected skin, which are a hallmark of neuropathic syndromes. This phenomenon is attributed to ‘central sensitization’: Enhanced transmission of non-nociceptive input to spinal dorsal horn neurons in the nociceptive pathway; transient noxious stimulation can temporarily induce this condition and heating capsaicin-treated skin is a human model. Applying this model to the patients with a loss-of-function mutation in the gene for PIEZO2 failed to cause tactile allodynia, consistent with their inability to detect light touch and vibration but in contrast to their largely retained perception of innocuous and noxious pressure [29, 163]. These results are in agreement with findings from conditional PIEZO2 knockout mice that showed major deficits in responsiveness to various weak mechanical stimuli but only minor reduction of responses to pin prick and pinch. Single-fibre recordings from C- and Aδ-nociceptors in the ex vivo saphenous nerve-skin preparation showed only reductions of the short dynamic phase of discharge evoked by noxious punctate force stimulation, the subsequent static discharge phase was the same in knockouts and wildtypes [116]. In the rat skin, only C- and Aδ-nociceptors but not low-threshold Aβ-mechanoreceptors were able to encode different noxious pressures over two minutes of constant stimulation, and only the high-threshold Aδ-fibres (HTM-Aδ) got markedly sensitized by such traumatic stimuli, which corresponds to mechanical hyperalgesia after pinching human skin folds [67, 140]. Similar myelinated high-threshold mechanoreceptors able to discriminate intensities of noxious mechanical stimuli have recently been found by human microneurography, but the patients with PIEZO2 loss-of-function were able to discriminate the stimuli that excited those nerve fibres [118].

Apart from low-threshold mechanoreceptive (LTM) Aβ-fibres that all express PIEZO2, this expression in LTM C-fibres has not safely been established [66]. These slowly adapting fibres have been shown to contribute to tactile allodynia in mouse models of inflammation, nerve injury, and trauma [56, 152]. Paradoxically, the same LTM C-fibres seem to mediate pleasant, rewarding touch in humans, whereas they also contribute to signalling pain in the rodent formalin test that is largely mediated by TRPA1 activation [99, 172]*.*

The other extreme in the skin is represented by the apparently mechanoinsensitive C-fibres (CM_i_ or C-MIA, ‘sleeping’ or ‘silent’ nociceptors) that respond vigorously to capsaicin and histamine and can be sensitized to mechanical stimulation by inflammatory mediators including nerve growth factor (NGF). CM_i_-fibres have been demonstrated in rat and non-human primate skin as well as by human microneurography [93, 122, 151, 183]. In mouse skin, CM_i_ have not safely been identified [73]. Recently, a novel biomarker for CM_i_ in mouse sensory neurons has been discovered, the nicotinic acetylcholine receptor subunit alpha-3 encoded by the CHRNA3 gene, and it was shown that the labelled DRG neurons project to visceral organs and other deep tissues but not to the skin. These initially mechanoinsensitive neurons (in primary culture) express PIEZO2 and become responsive to mechanical stimulation after prolonged treatment with an inflammatory mediator combination or with NGF [133]. This corroborates earlier work from Patapoutian’s lab showing that bradykinin, by activation of protein kinase A or C, potentiates mechanically evoked inward currents through PIEZO2 [46]. In addition, a neuronal upregulation of PIEZO2 immunoreactivity has recently been described that follows upon cutaneous inflammation, osteoarthritis, and in neuropathic pain models [153]. This may indicate an epigenetic upregulation of PIEZO2 expression that may not only ‘awaken sleeping’ nociceptors in viscera, but also enhance the mechanosensitivity of the ‘ordinary’ polymodals in the skin. Acute exposure of those mechano-heat-sensitive C-fibres to a combination of inflammatory mediators is not sufficient to sensitize them to mechanical stimulation, although it most effectively enhances heat responsiveness [150]. This suggests that a transcriptional upregulation is required to achieve sustained mechanical hypersensitivity as induced, for instance by axonal transport of NGF and activation of ERK1/2 regulating nuclear gene expression [133]. Microinjection of NGF in human skin induces delayed mechanical hyperalgesia lasting for up to seven weeks but also increases cold and heat sensitivity for up to three weeks, suggesting upregulation of several sensory transducers [146]. Consequently, several pharmaceutical companies have developed monoclonal antibodies against NGF and performed clinical trials on osteoarthritic pain, but none has yet been approved by FDA and EMA, perhaps because the drugs were ‘too good’, seducing patients to overload their worn-out joints with sportive activities which too often led to joint replacement surgeries [137].

Finally, a large discrepancy needs to be discussed that occurs between the DRG patch-clamp and the single-fibre or microneurographic recordings: The mechanically evoked depolarizing currents are all more or less rapidly adapting within a range of some hundreds of milliseconds. However, the discharge activity of ‘real’ nociceptors, i.e. cutaneous nerve endings, lasts as long as the noxious pressure is maintained following an initial dynamic response [67, 78]. This indicates a heterogeneity of mechanically evoked currents, which leaves room for the previously mentioned mechanosensitivities of other mechanisms of stretch activation. The discrepancy goes even further, considering that a constant noxious pinch of human skin causes increasing pain, while the nociceptors clearly show adaptation, though with hundreds of seconds time constant [2, 55, 67]. This contradiction may be resolved by recruitment of initially unaffected nociceptors through diffusing mediators (neuropeptides, arachidonic acid derivatives) released from the bruised skin. Indeed, HTM Aδ-fibres show delayed and *crescendo*-like discharge activity when skin just outside their receptive field is sustainably pinched [140]. This sensitizing mechanism may involve closing the K2P channels and/or activating the TRP channels which are chemosensors in the first place.

## Other discoveries of the laureates

The Nobel Prize has been awarded ‘for their discoveries of receptors for temperature and touch’. However, the laureates also have other achievements that are less well known. As a punctual and incomplete selection, David Julius has cloned the serotonin receptor 5-HT1c [84], the only ionotropic 5-HT3 serotonin receptor [104] and an ATP receptor [100]. He also addressed Gα protein coupling [40] before he turned his expression cloning expertise towards the discovery of TRP channels [30]. Several unusual channel functions have been elucidated by his affinity to toxins, not only modulating TRP channels [22] but also a coral snake toxin acting on acid-sensing ion channels [21], a tarantula toxin acting on NaV1.1 [124], a pit viper toxin causing ATP release [185], or a snail toxin inhibiting 5-HT3 receptors [53]. As a side note, papers considering electroception [14, 15], itch [49], but also the heterogeneity of mechanosensitive sensory neurons should be mentioned [20]. The latter topic leads to Ardem Patapoutian, who started with developmental biology. The first first-author papers consider muscle development in mice [129, 130] before turning to the development of sensory neurons. Here, wnt-based development through neurotrophin signalling [128] and TrkA and TrkC influence on neuronal fate should be mentioned [112]. The papers considering TRP channels dominate the publication list in the period 2002–2011 which switches to PIEZO channels thereafter. Exceptions are the contributions to the physiology of volume-regulated anion channels [86, 135, 161], although an association with mechanical activation could be argued.

## Translational relevance in medicine

For the few who suffer from a rare disease and undergo human genetic diagnostics, understanding the functions of the described ion channels allows to judge whether a genetic alteration fits to the observed phenotype. Several single nucleotide polymorphisms or other mutations with phenotypic consequence are already known. The time of individual gene editing will come; therefore, loss- or gain-of-function mutations with a severe phenotype in well-understood ion channels could, one day, prompt gene-therapeutic correction.

For the many other patients, new pharmacological options would be relevant. So far, these have not materialized, and this could perhaps never happen due to the widespread bodily functions of these targets. Despite extensive efforts to target the TRP channels, no TRP channel inhibitor is on the market; considering TRPV1, many drug development projects have been scrapped—perhaps overprotectively—due to adverse effects. Also, more recently developed compounds not increasing body temperature seem to lack efficacy, although this cannot be well judged as little is published about ‘failed’ trials. The PIEZOs are involved in so many physiological functions that it remains open whether there will be indications where intended effects would outweigh off-target effects. In case there were tissue-specific cofactors of PIEZO channels, these might also allow to target their function only in a particular tissue. Epidermal application of antagonists for patients with mechanical hypersensitivity could avoid systemic side effects.

Even at this time, when the milestones of scientific discovery contributed by the Nobel laureates have not manifested in therapeutic options for the population at large, the fundamental importance of knowing and understanding a physiological mechanism on which models of disease are resting can hardly be overstated.
