# Correction to: Nobel somatosensations and pain

**DOI:** 10.1007/s00424-022-02689-5

**Published:** 2022-04-05

**Authors:** Peter W. Reeh, Michael J. M. Fischer

**Affiliations:** 1 Institute of Physiology and Pathophysiology, University of Erlangen-Nürnberg, Universitätsstrasse 17, 91052 Erlangen, Germany; 2 Center for Physiology and Pharmacology, Medical University of Vienna, Schwarzspanierstrasse 17, 1090 Vienna, Austria

**Correction to** **:** **Pflügers Archiv—European Journal of Physiology (2022) 474:405–420**

10.1007/s00424-022-02667-x

The online version of the article contains an error. Reference #2 should be replaced by: Adriaensen H, Gybels J, Handwerker HO, Van Hees J (1984) Nociceptor discharges and sensations due to prolonged noxious mechanical stimulation—a paradox. Human Neurobiol 3: 53–58.

The original article has been corrected.

